# Multi‐Breed Genomic Predictions for Average Daily Gain in Three Italian Beef Cattle Breeds

**DOI:** 10.1111/jbg.70004

**Published:** 2025-07-15

**Authors:** Daniele Colombi, Renzo Bonifazi, Fiorella Sbarra, Andrea Quaglia, Mario P. L. Calus, Emiliano Lasagna

**Affiliations:** ^1^ Dipartimento di Scienze Agrarie, Alimentari e Ambientali University of Perugia Perugia Italy; ^2^ Animal Breeding and Genomics Wageningen University and Research Wageningen the Netherlands; ^3^ Associazione Nazionale Allevatori Bovini Italiani da Carne (ANABIC) Perugia Italy

**Keywords:** genomic selection, linear regression validation, local breeds, multi‐trait, ssGBLUP

## Abstract

Marchigiana, Chianina, and Romagnola are three Italian autochthonous beef cattle breeds that have been historically selected for meat production. Recent advancements suggest that the use of genomic data and multi‐breed (MB) models to combine information from different breeds may help to increase the accuracies of genomic predictions, in particular if the available data per breed is limited. This study aimed to evaluate and compare the accuracies of genomic predictions for average daily gain (ADG) in the three Italian breeds. We implemented different scenarios using phenotypes collected on 5303 young bulls in performance tests across the three breeds, 23,793 pedigree records, and 4593 genotypes, and then validated through the linear regression method. The implemented scenarios were: pedigree Best Linear Unbiased Prediction (pBLUP) and single‐step Genomic BLUP (ssGBLUP) single‐trait single‐breed evaluations where each breed was modelled separately; pBLUP and ssGBLUP single‐trait multi‐breed evaluations where ADG was modelled as the same trait for all breeds, and ssGBLUP multi‐trait multi‐breed evaluations where ADG was considered as a different correlated trait across breeds. In addition, single‐ and multi‐breed pBLUP and ssGBLUP evaluations were implemented including weight at 1 year of age and muscularity as correlated traits of ADG in a multi‐trait approach. Results highlighted the improved accuracies (an average of 5% in ssGBLUP models compared to corresponding pBLUP ones) when incorporating genomic data in the prediction models. Moreover, single‐trait multi‐breed scenarios resulted in higher accuracy for breeds with lower heritabilities for ADG (an average of 4% for single‐trait multi‐breed models compared to single‐breed ones), confirming the importance of leveraging data from populations with higher heritabilities. Lastly, adding two correlated traits next to ADG in the single‐ and multi‐breed ssGBLUP yielded even higher accuracies than the scenarios only encompassing ADG. The observed increases in accuracy when leveraging data from more populations and/or more traits could be helpful when implementing genomic predictions for innovative traits with limited records per individual or low heritabilities, and for the genetic improvement of local populations where limited data availability represents a challenge for traditional genetic selection.

## 1 Introduction

Marchigiana (MAR), Chianina (CHI), and Romagnola (ROM) are Italian autochthonous beef cattle breeds. Although they are relatively small and local populations, with about 50,000 live animals each for MAR and CHI, and 10,000 for ROM (ANABIC 2024), they represent an important part of the Italian beef cattle industry, tradition, and cultural heritage (Gargani et al. 2015). These breeds are farmed along the Apennine range in central Italy and their labelled meat is protected by geographical indication PGI, “Vitellone Bianco dell'Appennino Centrale” (European Commission 1998).

MAR, CHI, and ROM breeds all descend from the Podolian trunk (Maretto et al. 2012) and, despite decades of intensive selection within breed and specialisation for meat production, they maintained phenotypic and genetic similarity across the breeds (Colombi et al. 2024). The selection and management of the three breeds are performed by the National Association of Italian Beef Cattle Breeders (ANABIC) and a performance test on young bulls is conducted at its genetic station, where also the best animals are approved for artificial insemination and used for the genetic improvement of these populations (Sbarra, Dal Zotto, and Mantovani 2009). The main criteria used for selection are the animals' growth and muscularity (Sbarra, Mantovani, and Bittante 2009). Selection has been carried out using traditional pedigree‐based Best Linear Unbiased Prediction (pBLUP), while more recently single‐step Genomic Best Linear Unbiased Prediction (ssGBLUP) models have been implemented in a pilot phase. Finally, the current genetic evaluations model each breed individually.

To date, genomic selection in beef cattle remains less adopted compared to dairy. This is due to several factors that make genomic selection in beef cattle more challenging and less economically advantageous (Meuwissen et al. 2016; Esrafili Taze Kand Mohammaddiyeh et al. 2023; Hayes et al. 2023): the use of artificial insemination is limited (van Eenennaam et al. 2014); genomic selection requires large reference populations, while beef cattle usually are divided into more breeds of smaller population (Lund et al. 2014); meat‐related traits usually show higher heritabilities than milk traits, making them easier to select even without genomic data; and finally, breeding animals can be accurately selected after completing the performance test, and progeny testing is not usually required since many traits of interest (such as growth and weight) can be measured on the selection candidates themselves. Thus, a performance test already provides good accuracies. This is not the case with sex‐linked traits such as milk production, which requires a progeny test indeed. Moreover, genomic predictions have been found to be less accurate in beef compared to dairy cattle because of the smaller size of the reference populations, and the reduced relatedness between target and validation groups (Meuwissen et al. 2016). Nonetheless, genomic selection has more recently been implemented and proved to be extremely useful, helping and increasing the selection intensity and prediction accuracies for multiple traits, also in the beef industry (Berry et al. 2016; Magalhães et al. 2019; Fernandes Júnior et al. 2022; Miller 2022).

Multi‐breed (MB) reference populations have been proposed as a possible approach to solve the lack of data in small populations (Bolormaa et al. 2013; Lund et al. 2014; Porto‐Neto et al. 2015; Cardoso et al. 2021; Londoño‐Gil et al. 2024). At the same time, multi‐trait (MT) models have yielded higher accuracies for genomic predictions for traits with no records for selection candidates or low heritabilities (Guo et al. 2014; Haque et al. 2024), if selection candidates have records for genetically correlated traits also included in the evaluation (Calus and Veerkamp 2011). Thus, the aim of this work was to evaluate the feasibility of and to validate multi‐breed ssGBLUP genomic predictions for average daily gain (ADG) in young MAR, CHI, and ROM bulls in performance tests. To this end, several multi‐breed and multi‐trait scenarios were implemented and validated.

## 2 Materials and Methods

### 2.1 Data Available

**FIGURE 1:**
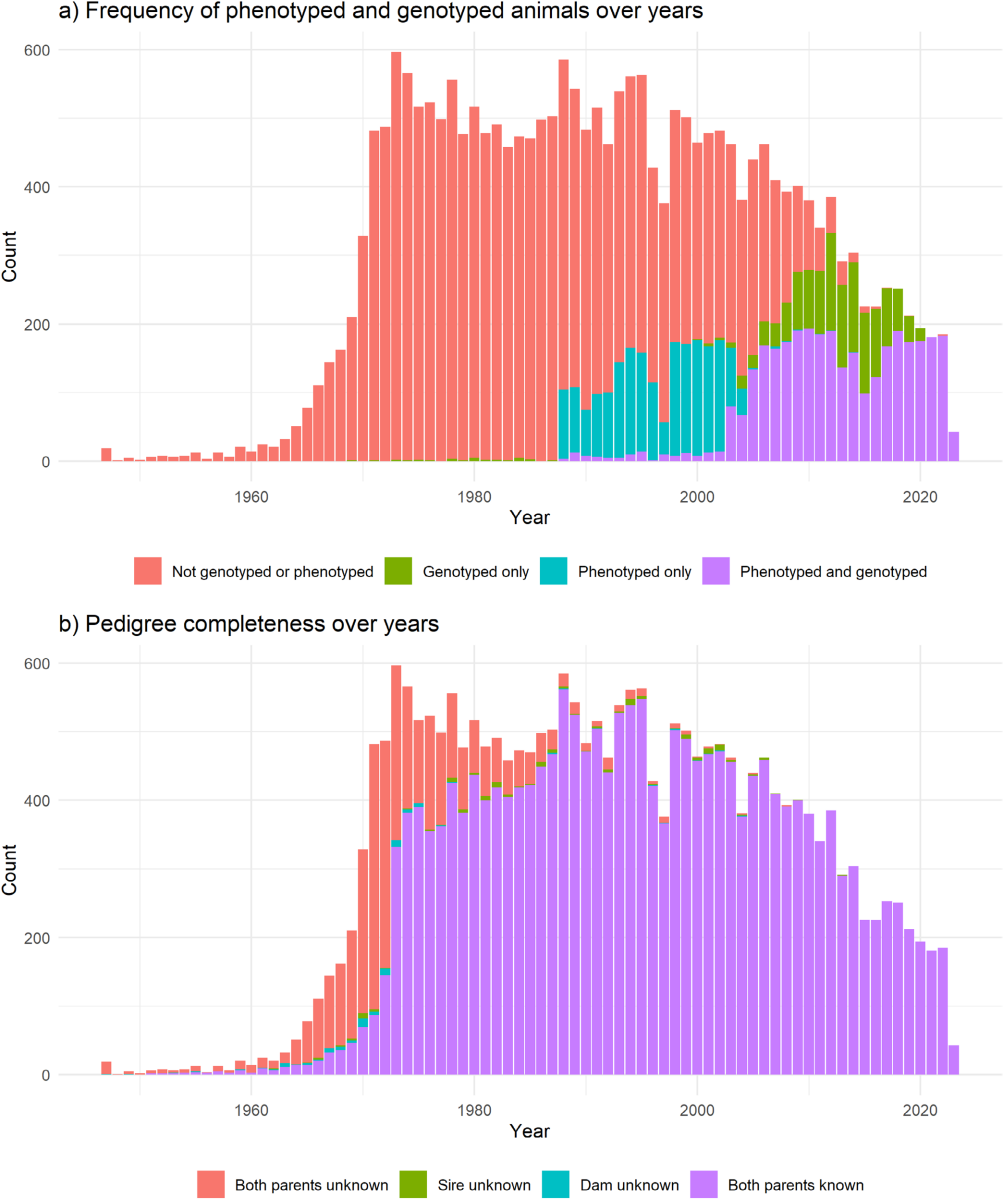
Distribution of animal data per year of birth. Within the total number of individuals in the pedigree, the following division, showed in a stacked manner, indicates: (a) how many animals are not genotyped or phenotyped (i.e., only pedigree information), genotyped only, phenotyped only, phenotyped and genotyped; (b) how many animals have both parents unknown, sire unknown, dam unknown, or both parents known. [Colour figure can be viewed at wileyonlinelibrary.com]

This study used a pedigree including 23,793 animals from all three breeds. Phenotypic records on ADG, muscularity, and weight at 1 year of age were available from 5303 young bulls in performance tests at ANABIC genetic station from 1988 to 2023. Young bulls are tested in a common genetic station for a 6‐month period, starting at an approximate age of 6 months (±1 month) and an average weight of 300 kg (Sbarra, Mantovani, and Bittante 2009; ANABIC home page 2024). The main investigated trait in our study was ADG during the performance test (kg/day). In two scenarios, weight at 1 year of age (i.e., at the end of performance test) (WEI, kg) and muscularity (MUS, score, evaluated as in Colombi et al. (2024)) were also used. Blood samples for genotyping were collected from the jugular veins of the young bulls at the end of the performance test period. Blood sampling was carried out by trained veterinarians, who adhered to standard procedures and relevant national guidelines to ensure appropriate animal care. The research was carried out in adherence to the guidelines and regulations outlined in the ARRIVE guidelines (https://arriveguidelines.org). Samples were collected in EDTA K3 coated vacuum tubes and stored at −20°C prior to use, and genomic DNA was extracted using the GenElute Blood Genomic DNA kit (Sigma Aldrich, St. Louis, MO, USA) according to the manufacturer's instructions. A total number of 4593 animals were genotyped with the GeneSeek Genomic Profiler Bovine LDv4 33 K chip (Illumina Inc., San Diego, CA, USA), which contains 30,111 SNPs, at the Agrotis Laboratory (LGS, Cremona, Italy) using standard multi‐sample protocols and reagents according to the manufacturer's instructions. A description of the dataset is reported in Table 1, while the distribution of pedigree, phenotypic, and genotypic records according to the year of birth is shown in Figure 1.

**TABLE 1:** Number of animals in the pedigree, with phenotypes, and with genotypes, and descriptive statistics for the three breeds.

Breed	Pedigree	Phenotypes	Genotypes	Phenotype + genotype	ADG (kg/d)			WEI (kg)			MUS (score)		
					μ (SD)	Min	Max	μ (SD)	Min	Max	μ (SD)	Min	Max
MAR	7694	1697	1563	1092	1.593 (0.238)	0.389	2.388	539.1 (51.3)	348.3	714.3	396.6 (64.7)	165.9	600
CHI	9022	1827	1546	1118	1.686 (0.235)	0.670	2.409	577.8 (51.2)	340.5	757.6	370.6 (61.4)	162.8	500
ROM	7077	1779	1484	1100	1.553 (0.221)	0.708	2.410	527.2 (53.6)	329.8	724.7	374.5 (56.8)	161.8	500
Total (MB)	23,793	5303	4593	3310	1.612 (0.238)	0.389	2.410	548.4 (56.7)	329.8	757.6	380.2 (62.0)	161.8	600

Abbreviations: ADG, average daily gain; CHI, Chianina; MAR, Marchigiana; MB, multi‐breed; MUS, muscularity; ROM, Romagnola; WEI, weight at 1 year of age.

### 2.2 Quality Control and Principal Component Analysis

Genotype quality control was performed with the BLUPF90 family of programs (Misztal et al. 2014) using default options and the following criteria: SNPs with call rates < 0.95, animals with call rates < 0.95, SNPs with minor allele frequencies < 0.05, and SNPs with Mendelian conflicts were removed. Quality control was both performed within each breed, as well as across all breeds combined. After quality control, genotypes from 1426 MAR at 22,454 markers, 1404 CHI at 21,667 markers, 1335 ROM at 21,942 markers, and 4165 animals at 23,038 markers for the multi‐breed scenarios were retained for further analysis. Principal Component Analysis (PCA) of the genotype data was performed using PreGSf90 software (Misztal et al. 2014).

### 2.3 Genomic Prediction Scenarios

Several scenarios were implemented to evaluate the increase in prediction accuracy with different single‐ and multi‐breed models and single‐ and multi‐trait approaches. The scenarios implemented are described below and Table 2 summarises the information used in each scenario.

- **SB_pBLUP**: Single‐trait single‐breed pedigree‐based best linear unbiased predictions (BLUP) with each breed evaluated separately.
- **SB_ssGBLUP**: Single‐trait single‐breed single‐step genomic BLUP (ssGBLUP) with each breed evaluated separately.
- **STMB_pBLUP**: Single‐trait multi‐breed pedigree‐based BLUP, in which ADG is considered as the same trait across breeds.
- **STMB_ssGBLUP**: Single‐trait multi‐breed single‐step GBLUP, in which ADG is considered as the same trait across breeds.
- **MTMB_ssGBLUP**: Multi‐trait multi‐breed single‐step GBLUP, in which ADG is considered as a different correlated trait across breeds.
- **MTMB_W_ssGBLUP**: Same as MTMB_ssGBLUP, but using an **H** matrix computed accounting for different allele frequencies within each breed according to Wientjes et al. (2017).
- **MTMB_MF_ssGBLUP**: Same as MTMB_ssGBLUP, but using one metafounder (MF) for each breed.

**TABLE 2:** Information used in each scenario: Whether the relationship matrix was built using only pedigree or pedigree and genotypes; whether a multi‐breed modelling approach was used; whether ADG was considered as the same or different trait in multi‐breed models; and whether WEI and MUS were included in the model.

Scenario	Pedigree	Genotypes	Multi‐breed	ADG as different trait across breeds	WEI + MUS
SB_pBLUP	●				
SB_ssGBLUP	●	●			
STMB_pBLUP	●		●		
STMB_ssGBLUP	●	●	●		
MTMB_ssGBLUP	●	●	●	●	
MTMB_W_ssGBLUP	●	●	●	●	
MTMB_MF_ssGBLUP	●	●	●	●	
SB_3pheno_pBLUP	●				●
SB_3pheno_ssGBLUP	●	●			●
MB_3pheno_ssGBLUP	●	●	●		●

Abbreviations: ADG, average daily gain; MUS, muscularity; WEI, weight at 1 year of age.

Additionally, two scenarios were implemented to evaluate differences and quantify the possible advantages in the accuracy of genomic predictions for ADG when including additional correlated traits. In these two scenarios, WEI and MUS were correlated to ADG using a multi‐trait approach.

- **SB_3pheno_pBLUP**: Single‐breed pedigree‐based BLUP with each breed evaluated separately, and including WEI and MUS data next to ADG using a multi‐trait approach.
- **SB_3pheno_ssGBLUP**: Single‐breed single‐step GBLUP with each breed evaluated separately, and including WEI and MUS data next to ADG using a multi‐trait approach.
- **MB_3pheno_ssGBLUP**: Multi‐breed single‐step GBLUP with MAR, CHI, and ROM combined in a joint multi‐breed population, and including WEI and MUS data next to ADG using a multi‐trait approach.

For all the genomic prediction scenarios, the **G** matrix was constructed according to method 1 described by van Raden (2008). For the STMB scenarios, breeds were merged followed by construction of the matrix without further breed‐specific adjustments. For the MTMB scenarios, we applied three different strategies: a baseline multi‐breed multi‐trait model implemented in the Blupf90+ suite (Misztal et al. 2014) with no breed‐specific scaling (MTMB_ssGBLUP); an adjustment by scaling **G** according to breed‐specific allele frequencies as described by Wientjes et al. (2017) in the MTMB_W_ssGBLUP scenario; finally, a blended **G** matrix was used in combination with metafounders in the MTMB_MF_ssGBLUP scenario, as described in Legarra et al. (2015). All genomic scenarios implemented a single‐step approach, thus the **G** matrix was blended with the pedigree relationship matrix **A** in a combined relationship matrix **H**, following Aguilar et al. (2010), using default parameters as follows:

$$H^{-1}=A^{-1}+\left[\begin{array}{cc} 0 & 0\\ 0 & {\left(0.95G+0.05A_{22}\right)}^{-1}-A_{22}^{-1} \end{array}\right]$$

### 2.4 Statistical Models

This section describes the statistical models used when implementing the above scenarios.

The base model applied for SB_pBLUP, SB_ssGBLUP, STMB_pBLUP, and STMB_ssGBLUP scenarios was the following:

y=Xb+Zu+e

where **y** is the vector of phenotypes, **b** is the vector of fixed effects which included the contemporary groups (based on breed, month, and year of birth) and farm of origin, **u** is the vector of random additive genetic effects, **e** is the vector of random residual effects, **X** and **Z** are incidence matrices linking phenotypes to fixed and genetic additive effects, respectively. It was assumed that:

$$u\sim N\left(0,\sigma_{u}^{2}⊗K\right)$$

where $\sigma_{u}^{2}$ is the additive genetic variance for ADG, and **K** is the relationship matrix. The matrix **K** was a pedigree‐based relationship matrix (**A**) in the pBLUP scenarios and a combined pedigree and genomic relationship matrix (**H**) in the ssGBLUP scenarios (Aguilar et al. 2010; Mäntysaari et al. 2020). Residuals were assumed to be uncorrelated, i.e., **e** ~ *N*(0, $\sigma_{e}^{2}⊗$ **I**), where **I** is an identity matrix.

For the multi‐breed scenarios where ADG was considered as a different correlated trait across breeds, the model applied was the following:

$$\left[\begin{array}{c} y_{\mathrm{MAR}}\\ y_{\mathrm{CHI}}\\ y_{\mathrm{ROM}} \end{array}\right]=\left[\begin{array}{ccc} X_{\mathrm{MAR}} & 0 & 0\\ 0 & X_{\mathrm{CHI}} & 0\\ 0 & 0 & X_{\mathrm{ROM}} \end{array}\right]\;\left[\begin{array}{c} b_{\mathrm{MAR}}\\ b_{\mathrm{CHI}}\\ b_{\mathrm{ROM}} \end{array}\right]+\left[\begin{array}{ccc} Z_{\mathrm{MAR}} & 0 & 0\\ 0 & Z_{\mathrm{CHI}} & 0\\ 0 & 0 & Z_{\mathrm{ROM}} \end{array}\right]\;\left[\begin{array}{c} u_{\mathrm{MAR}}\\ u_{\mathrm{CHI}}\\ u_{\mathrm{ROM}} \end{array}\right]+\left[\begin{array}{c} e_{\mathrm{MAR}}\\ e_{\mathrm{CHI}}\\ e_{\mathrm{ROM}} \end{array}\right]$$

and it was assumed that:

$$\mathrm{Var}\;\left[\begin{array}{c} u_{\mathrm{MAR}}\\ u_{\mathrm{CHI}}\\ u_{\mathrm{ROM}} \end{array}\right]=G⊗H=\left[\begin{array}{ccc} \sigma_{u_{\mathrm{MAR}}}^{2} & & \mathrm{sym}\\ \sigma_{u_{\mathrm{MAR},\mathrm{CHI}}} & \sigma_{u_{\mathrm{CHI}}}^{2} & \\ \sigma_{u_{\mathrm{MAR},\mathrm{ROM}}} & \sigma_{u_{\mathrm{CHI},\mathrm{ROM}}} & \sigma_{u_{\mathrm{ROM}}}^{2} \end{array}\right]⊗H$$

where **G** is the genetic co‐variance matrix; $\sigma_{u_{\mathrm{MAR}}}^{2}$, $\sigma_{u_{\mathrm{CHI}}}^{2}$, and $\sigma_{u_{\mathrm{ROM}}}^{2}$ are the genetic variances for ADG in MAR, CHI, and ROM; $\sigma_{u_{\mathrm{MAR},\mathrm{CHI}}}$, $\sigma_{u_{\mathrm{MAR},\mathrm{ROM}}}$, and $\sigma_{u_{\mathrm{CHI},\mathrm{ROM}}}$ are the genetic covariances between breeds. Residuals were assumed to be uncorrelated across breeds.

In the scenarios including WEI and MUS, the model applied was the following:

$$\left[\begin{array}{c} y_{\mathrm{ADG},i}\\ y_{\mathrm{WEI},i}\\ y_{\mathrm{MUS},i} \end{array}\right]=\left[\begin{array}{ccc} X_{\mathrm{ADG},i} & 0 & 0\\ 0 & X_{\mathrm{WEI},i} & 0\\ 0 & 0 & X_{\mathrm{MUS},i} \end{array}\right]\;\left[\begin{array}{c} b_{\mathrm{ADG},i}\\ b_{\mathrm{WEI},i}\\ b_{\mathrm{MUS},i} \end{array}\right]+\left[\begin{array}{ccc} Z_{\mathrm{ADG},i} & 0 & 0\\ 0 & Z_{\mathrm{WEI},i} & 0\\ 0 & 0 & Z_{\mathrm{MUS},i} \end{array}\right]\;\left[\begin{array}{c} u_{\mathrm{ADG},i}\\ u_{\mathrm{WEI},i}\\ u_{\mathrm{MUS},i} \end{array}\right]+\left[\begin{array}{c} e_{\mathrm{ADG},i}\\ e_{\mathrm{WEI},i}\\ e_{\mathrm{MUS},i} \end{array}\right]$$

where **y** is the vector of phenotypes (ADG, WEI, MUS) for breed *i* (MAR, CHI, ROM, or the multi‐breed population in the MB_3pheno_ssGBLUP scenario); **b**_*i*_ is the vector of fixed effects and covariates including for all traits the contemporary groups (as defined above) and the farm of origin, alongside weight at the beginning of performance test and age at measurement, as covariates, for WEI and MUS, respectively; other terms are defined as above. It was assumed that:

$$\mathrm{Var}\;\left[\begin{array}{c} u_{\mathrm{ADG},i}\\ u_{\mathrm{WEI},i}\\ u_{\mathrm{MUS},i} \end{array}\right]=G⊗K=\left[\begin{array}{ccc} \sigma_{u_{\mathrm{ADG},i}}^{2} & & \mathrm{sym}\\ \sigma_{u_{\mathrm{ADG},\mathrm{WEI},i}} & \sigma_{u_{\mathrm{WEI},i}}^{2} & \\ \sigma_{u_{\mathrm{ADG},\mathrm{MUS},i}} & \sigma_{u_{\mathrm{WEI},\mathrm{MUS},i}} & \sigma_{u_{\mathrm{MUS},i}}^{2} \end{array}\right]⊗K$$

where **K** was a pedigree‐based relationship matrix (**A**) in the pBLUP scenario and a combined pedigree and genomic relationship matrix (**H**) in the ssGBLUP scenarios; $\sigma_{u_{\mathrm{ADG},i}}^{2}$, $\sigma_{u_{\mathrm{WEI},i}}^{2}$, and $\sigma_{u_{\mathrm{MUS},i}}^{2}$ are the genetic variances for ADG, WEI, and MUS for breed *i* (MAR, CHI, ROM, or the multi‐breed population in the MB_3pheno_ssGBLUP scenario); $\sigma_{u_{\mathrm{ADG},\mathrm{WEI},i}}$, $\sigma_{u_{\mathrm{ADG},\mathrm{MUS},i}}$, and $\sigma_{u_{\mathrm{WEI},\mathrm{MUS},i}}$ are the genetic covariances between the breeds; other terms are defined as above. Residuals were assumed to be uncorrelated within and across breeds.

### 2.5 Variance Components Estimation (VCE)

Within each scenario, variance components and heritabilities (*h* ^2^) were estimated by Gibbs sampling with the Gibbsf90+ software (Misztal et al. 2014) using 100,000 cycles, 5000 cycles of burn‐in, and a thinning interval of 10. For the MTMB_ssGBLUP and MTMB_W_ssGBLUP scenarios, a total number of 200,000 cycles and 100,000 cycles of burn‐in were applied, with the same thinning interval of 10. For these two scenarios, only phenotyped animals with a genotype were retained for VCE, avoiding the inclusion of phenotypes not connected between breeds by neither pedigree nor genotype. This subsetting was applied only for VCE, and the full dataset was used for the estimation of genomic breeding values. The **H** matrix in the MTMB_W_ssGBLUP scenario was computed using calc_grm software (Calus and Vandenplas 2016). The **Γ** matrix for the MTMB_MF_ssGBLUP scenario was also computed using calc_grm, and it was defined as follows (Legarra et al. 2015):

$$\Gamma =\left[\begin{array}{ccc} \gamma_{\mathrm{MAR}} & & \mathrm{sym}\\ \gamma_{\mathrm{MAR},\mathrm{CHI}} & \gamma_{\mathrm{CHI}} & \\ \gamma_{\mathrm{MAR},\mathrm{ROM}} & \gamma_{\mathrm{CHI},\mathrm{ROM}} & \gamma_{\mathrm{ROM}} \end{array}\right]$$

where $\gamma_{\mathrm{MAR}}$, $\gamma_{\mathrm{CHI}}$, $\gamma_{\mathrm{ROM}}$ are the variances of the metafounders for each breed, and $\gamma_{\mathrm{MAR},\mathrm{CHI}}$, $\gamma_{\mathrm{MAR},\mathrm{ROM}}$, $\gamma_{\mathrm{CHI},\mathrm{ROM}}$ are the covariances across metafounders. The **Γ** matrix is reported in Table S1. The genetic variances were then scaled according to *k* = $\left(1+\frac{\bar{\mathrm{diag}\left(\Gamma \right)}}{2}-\bar{\Gamma }\right)$ (Legarra et al. 2015) with the Blupf90+ software (Misztal et al. 2014). VCE results were graphically inspected with Postgibbsf90 software to assess convergence (Misztal et al. 2014).

### 2.6 Estimation of Genomic Breeding Values and Validation

Pedigree‐based and single‐step genomic estimated breeding values (EBVs) were estimated using Blupf90+ software (Misztal et al. 2014) with default settings. EBVs were rebased according to a base population defined as phenotyped bulls born in 1990 and 1991 and composed of 55 MAR, 62 CHI, and 56 ROM individuals.

Pedigree and genomic predictions were validated using the linear regression method (LR; Legarra and Reverter (2018)). The LR method is a forward‐in‐time validation method that compares the EBV of a validation group called “focal group” from a “whole” evaluation (${\hat{u}}_{w}$) with the EBV from a “partial” evaluation (${\hat{u}}_{p}$) in which phenotypes after a specific time point are set to missing. Here, the phenotypes of animals born in the last ~4.5 years (considered as the average generation interval (Miller 2022)) were set to missing in the partial evaluation, i.e., animals born from January 2019 up to March 2023 (when the last data was available) were set to missing. The focal group was defined within each breed as the bulls born after the cut‐off and consisted of 262 MAR, 252 CHI, and 242 ROM individuals. Values of accuracy of partial EBV, dispersion bias with an expectation of 1 and values within 15% considered as acceptable, and level bias in genetic standard deviations (GSD), with an expectation of 0, were estimated using R functions provided in Bonifazi (2023) as follows (Bonifazi et al. 2022; Bonifazi 2023):

- Accuracy of partial EBV (${\hat{\mathrm{acc}}}_{p}$) = $\sqrt{\frac{\mathrm{cov}\left({\hat{u}}_{w},{\hat{u}}_{p}\right)}{\left(1-\bar{F}\right)\sigma_{u}^{2}}}$;
- Dispersion bias (${\hat{b}}_{p}$) = $\frac{\mathrm{cov}\left({\hat{u}}_{w},{\hat{u}}_{p}\right)}{\mathrm{var}\left({\hat{u}}_{p}\right)}$;
- Level bias in GSD (${\hat{\Delta }}_{p}$) = $\frac{\bar{\hat{u_{p}}}-\bar{\hat{u_{w}}}}{\sqrt{\sigma_{u}^{2}}}$,

where $\bar{F}$ is the mean inbreeding coefficient for the focal group, $\sigma_{u}^{2}$ is the additive genetic variance, $\bar{\hat{u_{p}}}$ and $\bar{\hat{u_{w}}}$ are the mean EBV of the focal group for the partial and whole evaluations, respectively. Finally, standard errors of the validation metrics were obtained using bootstrapping and 10,000 samples.

## 3 Results

### 3.1 Genetic Parameters

In line with Colombi et al. (2024), the first principal component (Figure S1) separated CHI from ROM; MAR was in‐between CHI and ROM according to the first component and further discriminated from the other two breeds by the second principal component.

**FIGURE 2:**
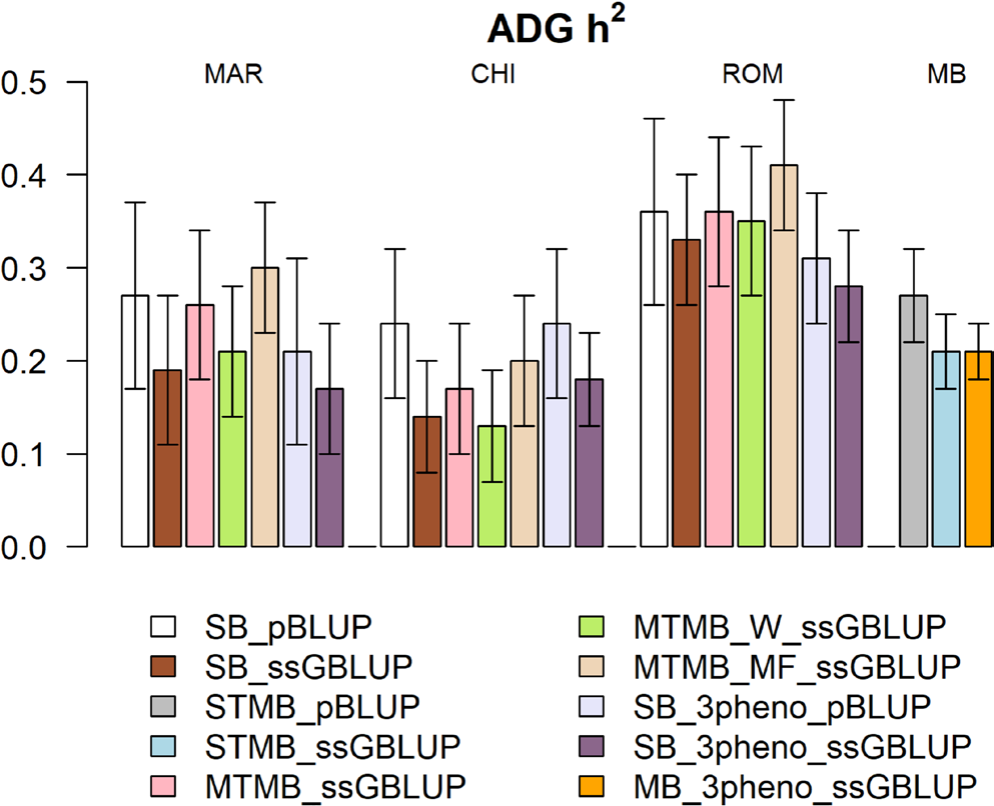
Estimated heritabilities. All values are reported within each breed for each scenario. Error bars represent standard errors. CHI, Chianina; MAR, Marchigiana; MB, Multi‐breed population including animals from MAR, CHI and ROM for those scenarios where ADG is considered as the same trait across all three breeds; ROM, Romagnola. [Colour figure can be viewed at wileyonlinelibrary.com]

Estimated heritabilities (*h* ^2^) for ADG within each breed and scenario are reported in Figure 2, while estimated genetic and residual variances are in Table S2. Estimated *h* ^2^ for MAR ranged from 0.17 to 0.30 according to the scenario. The estimated *h* ^2^ for CHI was the lowest across all breeds (ranging from 0.13 to 0.24), while ROM had considerably higher *h* ^2^ compared to the other breeds (ranging from 0.28 to 0.41).

Between‐breed genetic correlations for ADG were estimated using MTMB_ssGBLUP and MTMB_W_ssGBLUP models (Table S3). The estimates obtained with both models had large standard errors that did not allow for a correct interpretation of the true underlying genetic correlations between breeds. Respectively for MTMB_ssGBLUP and MTMB_W_ssGBLUP, the between‐breed genetic correlations for ADG were inconsistent between MAR and CHI with values (standard errors in parenthesis) of 0.45 (0.51) and −0.52 (0.23). The correlations between MAR and ROM (0.16 (0.61) and 0.61 (0.20)), and between CHI and ROM (0.39 (0.30) and 0.28 (0.31)), were more consistent between both models.

The within‐breed estimated genetic correlations between ADG and WEI were close to unity for all breeds ranging from 0.94 to 0.96 (0.001–0.002), depending on the breed and scenario for the ssGBLUP models (i.e., SB_3pheno_ssGBLUP or MB_3pheno_ssGBLUP), those correlations resulted lower in the pBLUP scenario (0.75–0.99 (0.02–0.20)). In the SB_3pheno_ssGBLUP scenario, for MAR and ROM, respectively, moderate genetic correlations for the ssGBLUP scenarios were estimated between ADG and MUS (0.35 (0.20) and 0.59 (0.10)), and between WEI and MUS (0.44 (0.17) and 0.43 (0.12)). The corresponding genetic correlations for CHI were considerably lower (0.17 (0.18) and 0.18 (0.15) for ADG‐MUS and WEI‐MUS, respectively) (Table S4).

### 3.2 Validation

#### 3.2.1 Accuracy of Partial EBV

**FIGURE 3:**
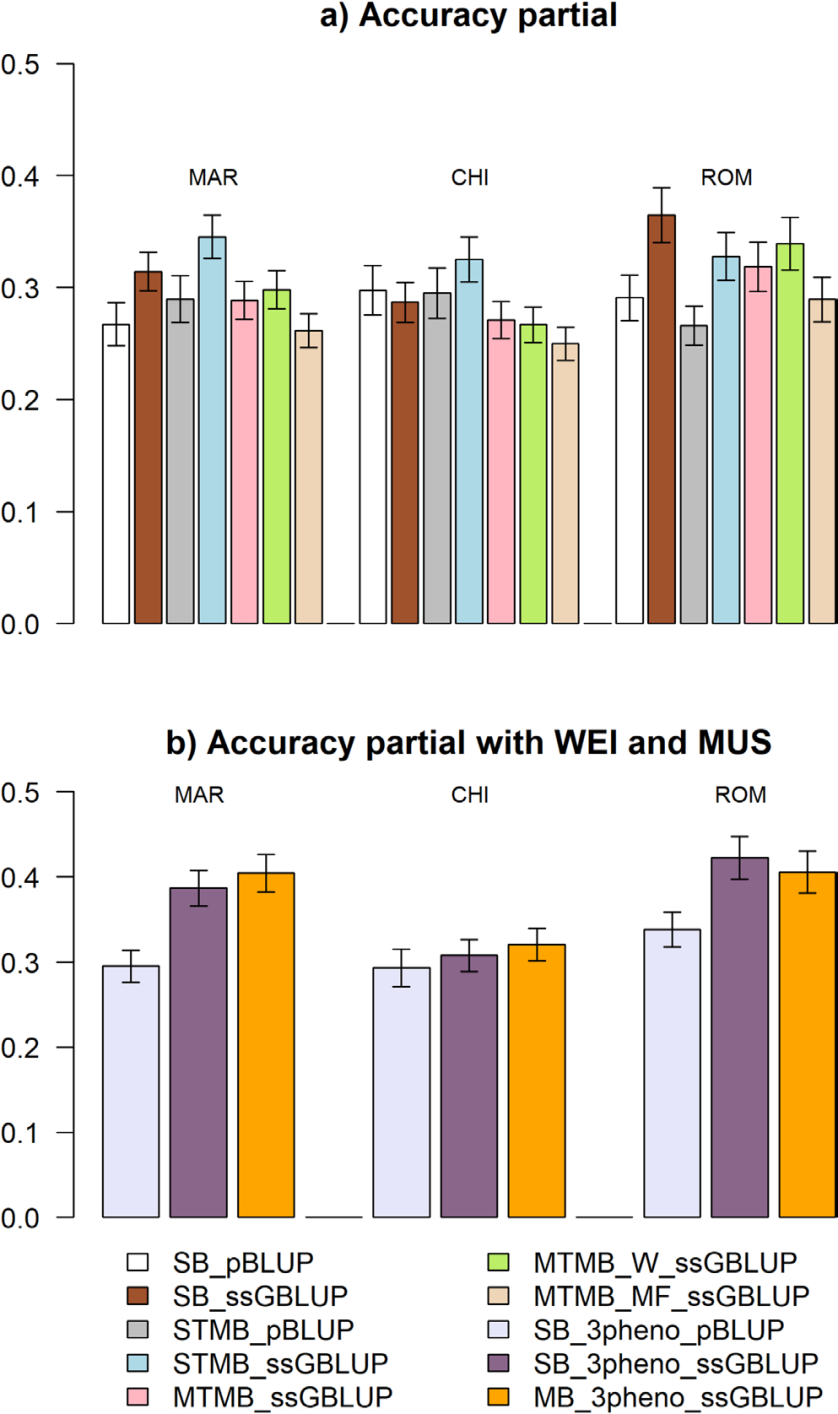
Accuracy of partial EBVs for each breed and scenario: (a) accuracies for scenarios in which only ADG was considered in the model; (b) accuracies for scenarios in which ADG was considered next to WEI and MUS in the model. CHI, Chianina; MAR, Marchigiana; ROM, Romagnola. Error bars represent standard errors. [Colour figure can be viewed at wileyonlinelibrary.com]

The ${\hat{\mathrm{acc}}}_{p}$ (Figure 3a,b and Table S5) was generally the highest for ROM (ranging from 0.27 to 0.42 across scenarios), followed by MAR (from 0.26 to 0.40) and CHI (from 0.25 to 0.33). Compared to traditional pBLUP, ssGBLUP evaluations strongly increased accuracy of predictions in all scenarios and breeds, especially for ROM. The only exception was for CHI which showed a slightly lower ${\hat{\mathrm{acc}}}_{p}$ in SB_ssGBLUP compared to SB_pBLUP (0.29 vs. 0.30). The STMB_ssGBLUP scenario resulted in the highest ${\hat{\mathrm{acc}}}_{p}$ for MAR and CHI (0.35 and 0.33, respectively), while for ROM, the SB_ssGBLUP scenario showed the highest ${\hat{\mathrm{acc}}}_{p}$ (0.37) (Figure 3a). Scenarios that also included WEI and MUS, generally resulted in much higher ${\hat{\mathrm{acc}}}_{p}$, compared to the corresponding scenarios with ADG only, for MAR and ROM (accuracies ranging from 0.34 to 0.42), due to the inclusion of more data and the genetic correlations between traits. CHI was an exception to this, since the SB_3pheno_pBLUP, SB_3pheno_ssGBLUP, and MB_3pheno_ssGBLUP scenarios resulted in slightly lower ${\hat{\mathrm{acc}}}_{p}$ than the STMB_ssGBLUP scenario (0.29, 0.31, and 0.32, respectively, vs. 0.33), most likely because of the close to zero genetic correlations between MUS and the other traits in this breed. Similarly to the scenarios were only ADG was used, the multi‐trait ssGBLUP evaluations yielded higher ${\hat{\mathrm{acc}}}_{p}$ compared to SB_3pheno_pBLUP model. Finally, for MAR and CHI, MB_3pheno_ssGBLUP resulted in somewhat higher accuracies compared to SB_3pheno_ssGBLUP (0.41 and 0.32, respectively, vs. 0.39 and 0.31) while for ROM the SB_3pheno_ssGBLUP model still had slightly higher accuracy compared to MB_3pheno_ssGBLUP model (0.42 vs. 0.41) (Figure 3b).

#### 3.2.2 Biases

**FIGURE 4:**
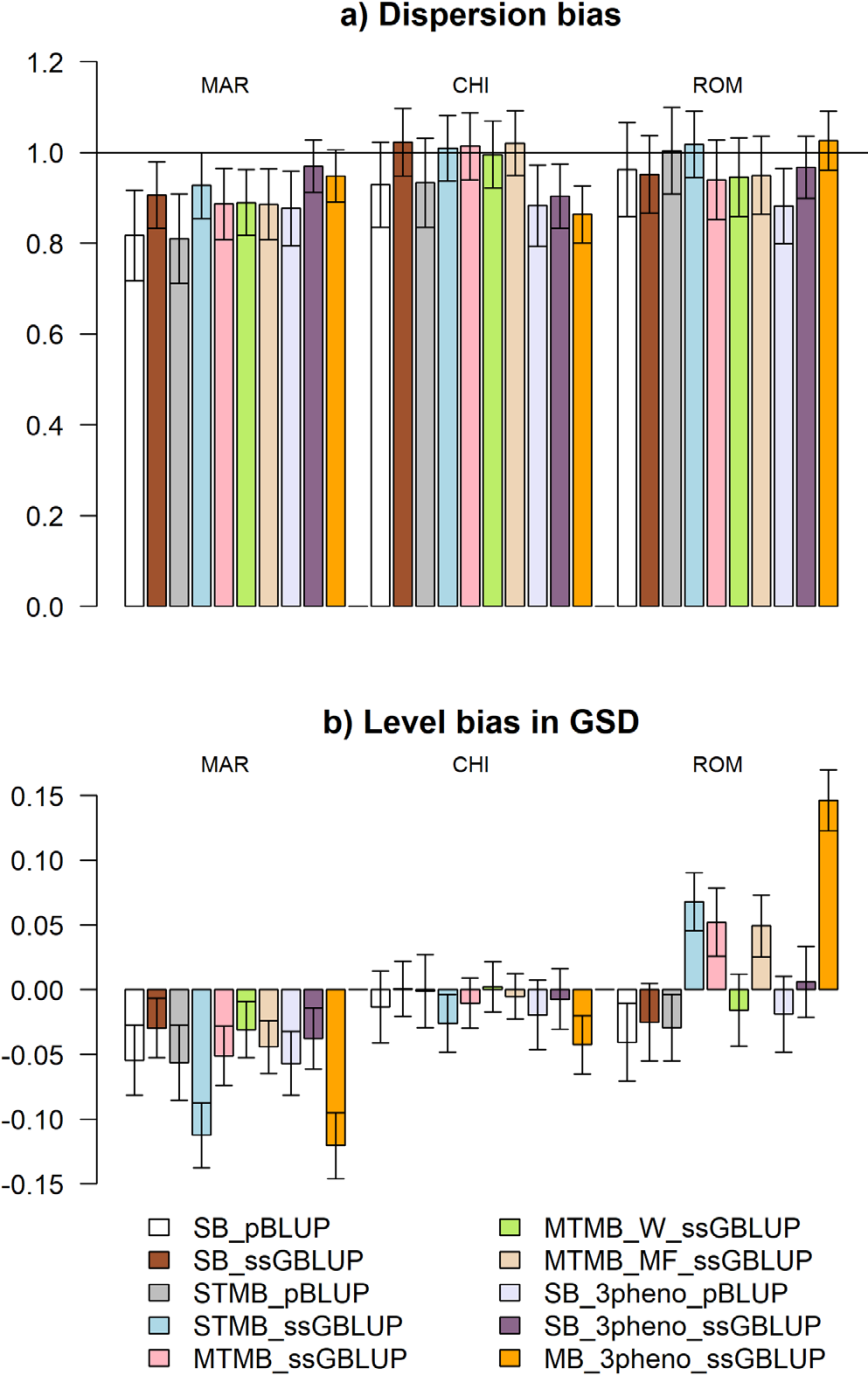
(a) dispersion bias (${\hat{b}}_{p}$). (b) level bias in genetic standard deviations (GSD) (${\hat{\Delta }}_{p}$). CHI, Chianina; MAR, Marchigiana; ROM, Romagnola. Error bars represent standard errors. [Colour figure can be viewed at wileyonlinelibrary.com]

Overall, for both dispersion bias and level bias (Figure 4a,b and Table S5), CHI showed the least dispersed and bias genomic predictions (i.e., values of ${\hat{b}}_{p}$ close to 1, and ${\hat{\Delta }}_{p}$ close to 0). All dispersion biases were in the acceptable range except for SB_pBLUP and STMB_pBLUP scenarios in MAR (0.817 and 0.810, respectively). In MAR, a negative level bias was observed in all scenarios, with the largest value of ${\hat{\Delta }}_{p}$ being −0.120 GSD in the MB_3pheno_ssGBLUP scenario. Regarding ROM, STMB_ssGBLUP, MTMB_ssGBLUP, MTMB_MF_ssGBLUP, and MB_3pheno_ssGBLUP scenarios showed positive level bias, with the MB_3pheno_ssGBLUP scenario showing the largest level bias (${\hat{\Delta }}_{p}$ of 0.146 GSD).

## 4 Discussion

This study implemented and validated several single‐ or multi‐breed and single or multi‐trait, pedigree‐based and single‐step genomic BLUP scenarios for ADG in three Italian beef cattle breeds. Moreover, three scenarios including weight at 1 year of age and muscularity as correlated traits were implemented to quantify the possible benefits for ADG when including additional correlated traits.

Principal component analysis was performed to evaluate the relationships and genetic similarity between the breeds. The PCA plot illustrated that ROM and CHI are immediately discriminated by PC1, being the most differentiated breeds among the Italian local beef cattle breeds (Colombi et al. 2024) while MAR was separated by PC2 and was placed between CHI and ROM. The observed pattern for MAR follows the expectation, as the breed originated in the beginning of the 20th century by crossing the local cattle originating in the Marche region with CHI, and thereafter with ROM to reduce its dimensions (Di Lorenzo et al. 2018).

Validation results confirm the advantages of including genomic data in beef cattle breeding programmes. Indeed, for both MAR and ROM the single‐step scenarios yielded higher accuracies than the pedigree‐based scenarios, both when only ADG was considered in the model and when WEI and MUS were added. For CHI, ssGBLUP outperformed pBLUP in the STMB scenario, while SB_pBLUP showed a slightly higher accuracy than SB_ssGBLUP (only 0.01 higher). Nonetheless, the SB_pBLUP scenario resulted in more over‐dispersed EBV than SB_ssGBLUP, confirming the benefits of implementing a single‐step evaluation also in CHI. Over‐dispersion outside what was considered acceptable was only observed in pedigree‐based scenarios for MAR, while all the other models showed dispersion within the acceptable range of 15% (Tsuruta et al. 2011; Bonifazi et al. 2022). The results of this study are consistent with other studies suggesting that ssGBLUP increases prediction accuracies compared to traditional pBLUP models also for beef traits (Park et al. 2020; Mancin et al. 2021; Bonifazi et al. 2022; Haque et al. 2024).

Overall, the MB models with ADG considered as the same trait across the three breeds (STMB_ssGBLUP) performed the best for MAR and CHI. For CHI, the STMB_ssGBLUP was also more accurate than the scenarios where WEI and MUS were included as correlated traits. Only the STMB_pBLUP scenario did not show any considerable advantage compared to the SB_pBLUP scenarios for all breeds. This could be due to the lack of pedigree relationships between the three breeds; thus, the difference between the STMB_pBLUP and SB_pBLUP scenarios is that the variance components were estimated either across all three breeds or for each breed separately, respectively. Interestingly, the ssGBLUP multi‐breed scenarios did not increase the accuracies for ROM, in which the single‐breed evaluations performed better. The higher heritability for ADG in ROM may explain the increases in accuracies for MAR and CHI when data from ROM were included. On the contrary, data from MAR and CHI, having lower heritabilities, did not improve the accuracy for ROM. We thus noticed that multi‐breed evaluations can improve genomic predictions for breeds with lower heritabilities, as has also been reported by Cardoso et al. (2021). On the other hand, a lower accuracy in MB compared to SB evaluation was observed by Cesarani et al. (2022) for breeds that were less represented in the reference population. This decrease in accuracy appears when a multi‐breed reference population is dominated by some breeds, undermining reliabilities for smaller populations (van den Berg et al. 2020; Cesarani et al. 2022), unless the trait's heritability is high (van den Berg et al. 2020). Although the ROM population is overall smaller than the MAR and CHI populations (Sarti et al. 2019; ANABIC 2024), we did not observe such a decrease in accuracy in our results since the number of animals involved in our study and in the reference populations was comparable between the breeds.

The MT scenarios, where ADG was considered as different traits across the three breeds, performed generally the worst. The relatively low amount of genotypic data available and the disconnected pedigree between the three breeds may have been insufficient to connect the trait across breeds, leading to the poor performance of the MT models. This could be confirmed by the estimated genetic correlations for ADG between MAR and CHI under the MTMB models which were estimated to be in an opposite direction in MTMB_ssGBLUP and MTMB_W_ssGBLUP (0.45 vs. −0.52), and by the extremely high standard errors for such correlations. In a similar way, Bonifazi et al. (2024) noticed a decrease in accuracy for calving season days in a multi‐breed population of Holstein, Jersey, and crossbred animals using a similar multi‐breed approach as in this study, although they also found an increase in accuracy for milk yield. The same reasons why MTMB_ssGBLUP and MTMB_W_ssGBLUP scenarios showed lower accuracy than the ST models might apply to the MTMB_MF_ssGBLUP scenario, whose variance components were retrieved from the MTMB_ssGBLUP scenario and scaled according to the **Γ** matrix. In conclusion, an assumed genetic correlation of one between the breeds in our data, as done in the ST models, gave the best results in terms of accuracy of genomic predictions. Moreover, the adjustments of the **G** matrix in the MTMB_W_ssGBLUP and MTMB_MF_ssGBLUP scenarios was not beneficial for the multi‐breed modelling of the three breeds, especially the implementation of three metafounders, which yielded the lowest accuracies across all the MB scenarios.

Overall, the scenarios including all three traits (ADG, WEI, MUS) performed the best for MAR and ROM, likely because of the higher genetic correlations between traits. The same was not true for CHI because a lower correlation between MUS and the other traits was observed. Contrary to the other breeds, ROM showed higher accuracies in the single‐breed model compared to the multi‐breed when all three traits were considered. The higher accuracies in the SB models are likely due to the higher *h* ^2^ of ADG in ROM, as already mentioned for the scenarios where only ADG was considered (Figure 3b). Tang et al. (2024), using simulated data, also observed that the best improvement for multi‐trait genomic predictions were obtained for low heritability traits which benefitted the most when correlated and high‐heritability traits were used in the model. Thus, genomic prediction accuracies increased with multi‐breed or multi‐trait scenarios that included phenotypes from genetically correlated populations and/or different traits compared to genomic evaluations using only single‐trait phenotypes or breeds, assuming that genetic correlations exist between such traits (Wang et al. 2023). More specifically, data from highly heritable and correlated traits are expected to be particularly beneficial when selecting for innovative, behavioural, or environmental traits, where the phenotyping cost is high leading to small amounts of phenotypic data available. In such situations, including genetically correlated traits in the genomic evaluation models may be used to improve the accuracies of EBV (Haque et al. 2024).

The results of this study, together with the routinely genotyping of young bulls in performance tests irrespective of the genomic‐based selection, confirm the favourable opportunity to move towards genomic selection in local beef cattle breeds, with potential benefits of implementing multi‐breed and multi‐trait models in genetic evaluations. For the Italian local breeds analysed in this study, more accurate genomic predictions could lead to quicker and more accurate choices of calves to admit for the performance test, accelerating their genetic progress. Moreover, implementing genomic predictions could improve selection for those breeds and traits where phenotyping records are scarce, the traits' heritability is low, or the reference population is small.

## 5 Conclusions

Different scenarios and models, both single‐ and multi‐breed, for genomic predictions of ADG in MAR, CHI, and ROM were evaluated. The results highlighted that ssGBLUP models improve the prediction accuracies for ADG, compared to traditional pBLUP models (0.34 vs. 0.29, respectively, on average across breeds). Multi‐breed evaluations that considered ADG in different breeds as the same trait resulted in higher accuracies for those breeds with lower heritabilities (MAR and CHI) while it did not benefit ROM, which had higher heritability for ADG. Lastly, multi‐trait scenarios that included two other beef traits were particularly beneficial for MAR and ROM due to their moderate genetic correlations between ADG and the other traits included, but not for CHI due to the low genetic correlations across traits. Thus, multi‐breed and multi‐trait models can be used for faster and more efficient selection by improving the accuracy of genomic predictions and thereby accelerating genetic improvement in smaller populations or local breeds that are challenging to breed with traditional approaches.

## Author Contributions

Conceptualization and methodology: D.C., R.B., M.P.L.C. Data curation and formal analysis: D.C., R.B. Writing – original draft preparation: D.C. Writing – review and editing: D.C., R.B., F.S., A.Q., M.P.L.C., E.L. Project supervision and administration: M.P.L.C., E.L. All authors have read and agreed to the final version of the manuscript.

## Conflicts of Interest

The authors declare no conflicts of interest.

## Supporting information


**Table S1:** Γ matrix of variances and covariances across metafounders for Marchigiana, Chianina, and Romagnola.
**Figure S1:** Plot of the first two principal components (PC) of the three breeds analysed.
**Table S2:** Estimated additive genetic variances ($\sigma_{u}^{2}$), residual variances ($\sigma_{e}^{2}$), and heritabilities ($h^{2}$) for ADG in every breed and scenarios. Standard errors are presented in parenthesis.
**Table S3:** Estimated genetic correlations and standard errors (in parenthesis) between the breeds in MTMB_ssGBLUP (below diagonal) and MTMB_W_ssGBLUP (above diagonal) for ADG.
**Table S4:** Estimated genetic correlations and standard errors (in parenthesis) between the traits in SB_3pheno_pBLUP (above the dotted line), SB_3pheno_ssGBLUP (below the dotted line) and MB_3pheno_ssGBLUP.
**Table S5:** Estimated accuracy of partial GEBV (${\hat{\mathrm{acc}}}_{p}$), dispersion bias (${\hat{b}}_{p}$), and level bias in GSD (${\hat{\Delta }}_{p}$) for ADG genomic predictions in every breed and scenarios.

## Data Availability

The raw phenotypic and genotypic data are stored in the drive cloud of the Department of Agricultural, Food and Environmental Sciences (DSA3) – University of Perugia and can be provided by the corresponding author on reasonable request.
