# Supplementary material for: Multi‐Breed Genomic Predictions for Average Daily Gain in Three Italian Beef Cattle Breeds
Source: J Anim Breed Genet. 2025 Jul 15;143(1):119–29. doi: 10.1111/jbg.70004 (PMC12686752; doi:10.1111/jbg.70004)
Supplement: Supplementary file 1 — Table S1: Γ matrix of variances and covariances across metafounders for Marchigiana, Chianina, and Romagnola. Figure S1: Plot of the first two principal components (PC) of the three breeds analysed. Table S2: Estimated additive genetic variances (σu2), residual variances (σe2), and heritabilities (h2) for ADG in every breed and scenarios. Standard errors are presented in parenthesis. Table S3: Estimated genetic correlations and standard errors (in parenthesis) between the breeds in MTMB_ssGBLUP (below diagonal) and MTMB_W_ssGBLUP (above diagonal) for ADG. Table S4: Estimated genetic correlations and standard errors (in parenthesis) between the traits in SB_3pheno_pBLUP (above the dotted line), SB_3pheno_ssGBLUP (below the dotted line) and MB_3pheno_ssGBLUP. Table S5: Estimated accuracy of partial GEBV (acc^p), dispersion bias (b^p), and level bias in GSD (Δ^p) for ADG genomic predictions in every breed and scenarios. [file JBG-143-119-s001.docx]

Supplementary Table S1: Γ matrix of variances and covariances across metafounders for Marchigiana, Chianina, and Romagnola.

**Γ =** $\left[ \begin{matrix} 0.400116 & & sym \\ 0.398505 & 0.490503 & \\ 0.363184 & 0.361602 & 0.417053 \end{matrix} \right]$


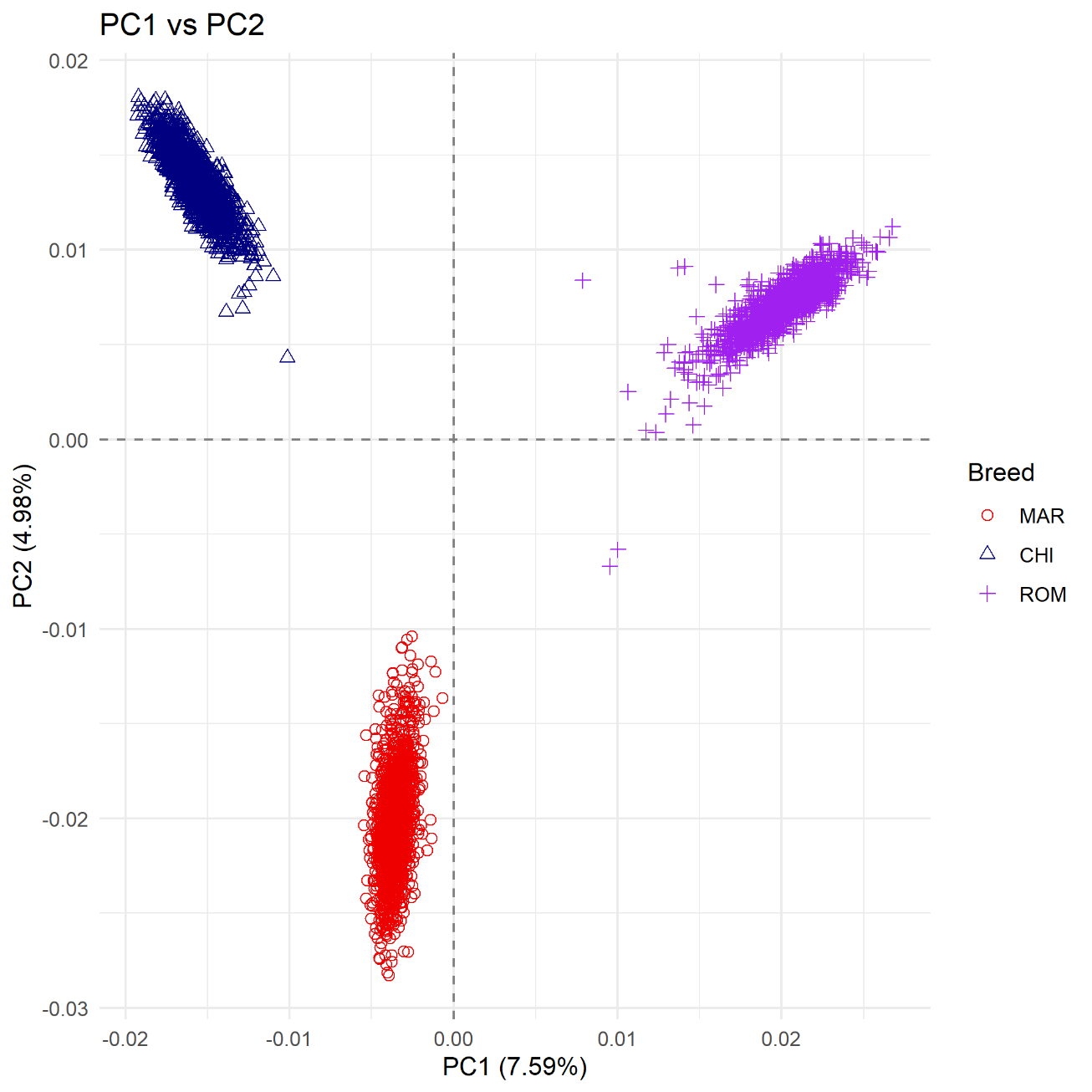


Figure S1: Plot of the first two principal components (PC) of the three breeds analysed.

Supplementary Table S2: Estimated additive genetic variances ($\sigma_{u}^{2}$), residual variances ($\sigma_{e}^{2}$), and heritabilities ($h^{2}$) for ADG in every breed and scenarios. Standard errors are presented in parenthesis.

|  | $\sigma_{u}^{2}$ | $\sigma_{e}^{2}$ | $h^{2}$ |
| --- | --- | --- | --- |
| **MAR** |  |  |  |
| SB_pBLUP | 0.0112 (0.0045) | 0.0296 (0.0037) | 0.27 (0.10) |
| SB_ssGBLUP | 0.0076 (0.0032) | 0.0325 (0.0029) | 0.19 (0.08) |
| MTMB_ssGBLUP | 0.0130 (0.0041) | 0.0368 (0.0038) | 0.26 (0.08) |
| MTMB_W_ssGBLUP | 0.0107 (0.0039) | 0.0401 (0.0041) | 0.21 (0.07) |
| MTMB_MF_ssGBLUP | 0.0158 (0.0050) | 0.0368 (0.0038) | 0.30 (0.07) |
| SB_3pheno_pBLUP | 0.0088 (0.0044) | 0.0320 (0.0037) | 0.21 (0.10) |
| SB_3pheno_ssGBLUP | 0.0068 (0.0030) | 0.0335 (0.0027) | 0.17 (0.07) |
| **CHI** |  |  |  |
| SB_pBLUP | 0.0078 (0.0026) | 0.0249 (0.0023) | 0.24 (0.08) |
| SB_ssGBLUP | 0.0044 (0.0021) | 0.0278 (0.0020) | 0.14 (0.06) |
| MTMB_ssGBLUP | 0.0067 (0.0030) | 0.0328 (0.0027) | 0.17 (0.07) |
| MTMB_W_ssGBLUP | 0.0050 (0.0025) | 0.0332 (0.0028) | 0.13 (0.06) |
| MTMB_MF_ssGBLUP | 0.0082 (0.0036) | 0.0328 (0.0027) | 0.20 (0.07) |
| SB_3pheno_pBLUP | 0.0079 (0.0029) | 0.0250 (0.0025) | 0.24 (0.08) |
| SB_3pheno_ssGBLUP | 0.0058 (0.0017) | 0.0269 (0.0017) | 0.18 (0.05) |
| **ROM** |  |  |  |
| SB_pBLUP | 0.0113 (0.0038) | 0.0201 (0.0029) | 0.36 (0.10) |
| SB_ssGBLUP | 0.0104 (0.0026) | 0.0206 (0.0020) | 0.33 (0.07) |
| MTMB_ssGBLUP | 0.0143 (0.0039) | 0.0250 (0.0028) | 0.36 (0.08) |
| MTMB_W_ssGBLUP | 0.0133 (0.0035) | 0.0248 (0.0028) | 0.35 (0.08) |
| MTMB_MF_ssGBLUP | 0.0174 (0.0047) | 0.0250 (0.0028) | 0.41 (0.07) |
| SB_3pheno_pBLUP | 0.0097 (0.0022) | 0.0216 (0.0019) | 0.31 (0.07) |
| SB_3pheno_ssGBLUP | 0.0087 (0.0020) | 0.0221 (0.0017) | 0.28 (0.06) |
| **Multi-breed as a single population** |  |  |  |
| STMB_pBLUP | 0.0091 (0.0019) | 0.0250 (0.0016) | 0.27 (0.05) |
| STMB_ssGBLUP | 0.0071 (0.0013) | 0.0270 (0.0012) | 0.21 (0.04) |
| MB_3pheno_ssGBLUP | 0.0071 (0.0013) | 0.0271 (0.0011) | 0.21 (0.03) |

ADG: average daily gain; MAR: Marchigiana, CHI: Chianina, ROM: Romagnola; scenarios are defined according to the manuscript and in Table 2.

Supplementary Table S3: Estimated genetic correlations and standard errors (in parenthesis) between the breeds in MTMB_ssGBLUP (below diagonal) and MTMB_W_ssGBLUP (above diagonal) for ADG.

|  | **MAR** | **CHI** | **ROM** |
| --- | --- | --- | --- |
| **MAR** | - | -0.52 (0.23) | 0.61 (0.20) |
| **CHI** | 0.45 (0.51) | - | 0.28 (0.31) |
| **ROM** | 0.16 (0.61) | 0.39 (0.30) | - |

ADG: average daily gain; MAR: Marchigiana, CHI: Chianina, ROM: Romagnola; scenarios are defined according to the manuscript and in Table 2.

Supplementary Table S4: Estimated genetic correlations and standard errors (in parenthesis) between the traits in SB_3pheno_pBLUP (above the dotted line), SB_3pheno_ssGBLUP (below the dotted line) and MB_3pheno_ssGBLUP.

|  | **MAR** | **CHI** | **ROM** | **MB** |
| --- | --- | --- | --- | --- |
| ADG-WEI | 0.78 (0.20) | 0.75 (0.16) | 0.99 (0.02) | 0.95 (0.04) |
|  | 0.94 (0.08) | 0.95 (0.05) | 0.96 (0.04) |  |
| ADG-MUS | 0.08 (0.43) | 0.23 (0.19) | 0.50 (0.14) | 0.32 (0.10) |
|  | 0.35 (0.20) | 0.17 (0.18) | 0.59 (0.10) |  |
| WEI-MUS | 0.36 (0.25) | 0.31 (0.19) | 0.50 (0.13) | 0.38 (0.09) |
|  | 0.44 (0.17) | 0.18 (0.15) | 0.43 (0.12) |  |

MAR: Marchigiana, CHI: Chianina, ROM: Romagnola; ADG: average daily gain; MAR: Marchigiana, CHI: Chianina, ROM: Romagnola; scenarios are defined according to the manuscript and in Table 2.

Supplementary Table S5: Estimated accuracy of partial GEBV ($\hat{acc}_{p}$), dispersion bias ($\hat{b}_{p}$), and level bias in GSD ($\hat{\Delta}_{p}$) for ADG genomic predictions in every breed and scenarios.

|  | Accuracy of partial GEBV ($\hat{acc}_{p}$) | Dispersion bias ($\hat{b}_{p}$) | Level bias in GSD ($\hat{\Delta}_{p}$) | |  |
| --- | --- | --- | --- | --- | --- |
| **MAR** |  |  | |  | |
| SB_pBLUP | 0.27 ± 0.02 | 0.82 ± 0.10 | | -0.05 ± 0.03 | |
| SB_ssGBLUP | 0.31 ± 0.02 | 0.91 ± 0.07 | | -0.03 ± 0.02 | |
| STMB_pBLUP | 0.29 ± 0.02 | 0.81 ± 0.10 | | -0.06 ± 0.03 | |
| STMB_ssGBLUP | 0.35 ± 0.02 | 0.93 ± 0.07 | | -0.11 ± 0.03 | |
| MTMB_ssGBLUP | 0.29 ± 0.02 | 0.89 ± 0.08 | | -0.05 ± 0.02 | |
| MTMB_W_ssGBLUP | 0.30 ± 0.02 | 0.89 ± 0.07 | | -0.03 ± 0.02 | |
| MTMB_MF_ssGBLUP | 0.26 ± 0.02 | 0.89 ± 0.08 | | -0.04 ± 0.02 | |
| SB_3pheno_pBLUP | 0.30 ± 0.02 | 0.88 ± 0.08 | | -0.06 ± 0.02 | |
| SB_3pheno_ssGBLUP | 0.39 ± 0.02 | 0.97 ± 0.06 | | -0.04 ± 0.02 | |
| MB_3pheno_ssGBLUP | 0.40 ± 0.02 | 0.95 ± 0.06 | | -0.12 ±0.03 | |
| **CHI** |  |  | |  | |
| SB_pBLUP | 0.30 ± 0.02 | 0.93 ± 0.09 | | -0.01 ± 0.03 | |
| SB_ssGBLUP | 0.29 ± 0.02 | 1.02 ± 0.07 | | 0.001 ± 0.21 | |
| STMB_pBLUP | 0.30 ± 0.02 | 0.93 ± 0.10 | | -0.001 ± 0.03 | |
| STMB_ssGBLUP | 0.33 ± 0.02 | 1.01 ± 0.07 | | -0.03 ± 0.02 | |
| MTMB_ssGBLUP | 0.27 ± 0.02 | 1.01 ± 0.07 | | -0.01 ± 0.02 | |
| MTMB_W_ssGBLUP | 0.27 ± 0.02 | 1.00 ± 0.07 | | 0.002 ± 0.02 | |
| MTMB_MF_ssGBLUP | 0.25 ± 0.02 | 1.02 ± 0.07 | | -0.01 ± 0.02 | |
| SB_3pheno_pBLUP | 0.29 ± 0.02 | 0.88 ± 0.09 | | -0.02 ± 0.03 | |
| SB_3pheno_ssGBLUP | 0.31 ± 0.02 | 0.90 ± 0.07 | | -0.01 ± 0.02 | |
| MB_3pheno_ssGBLUP | 0.32 ± 0.02 | 0.86 ± 0.06 | | -0.04 ± 0.02 | |
| **ROM** |  |  | |  | |
| SB_pBLUP | 0.29 ± 0.02 | 0.96 ± 0.10 | | -0.04 ± 0.03 | |
| SB_ssGBLUP | 0.37 ± 0.03 | 0.95 ± 0.09 | | -0.03 ± 0.03 | |
| STMB_pBLUP | 0.27 ± 0.02 | 1.00 ± 0.10 | | -0.02 ± 0.03 | |
| STMB_ssGBLUP | 0.33 ± 0.02 | 1.02 ± 0.07 | | 0.07 ± 0.02 | |
| MTMB_ssGBLUP | 0.32 ± 0.02 | 0.94 ± 0.09 | | 0.05 ± 0.03 | |
| MTMB_W_ssGBLUP | 0.34 ± 0.02 | 0.95 ± 0.09 | | -0.02 ± 0.03 | |
| MTMB_MF_ssGBLUP | 0.29 ± 0.02 | 0.95 ± 0.09 | | 0.05 ± 0.02 | |
| SB_3pheno_pBLUP | 0.34 ± 0.02 | 0.88 ± 0.08 | | -0.02 ± 0.03 | |
| SB_3pheno_ssGBLUP | 0.42 ± 0.03 | 0.97 ± 0.07 | | 0.01 ± 0.03 | |
| MB_3pheno_ssGBLUP | 0.41 ± 0.03 | 1.03 ± 0.07 | | 0.15 ± 0.02 | |

MAR: Marchigiana, CHI: Chianina, ROM: Romagnola; scenarios are defined according to the manuscript and in Table 2.
